# Association between gut microbiota dysbiosis and poor functional outcomes in acute ischemic stroke patients with COVID-19 infection

**DOI:** 10.1128/msystems.00185-24

**Published:** 2024-05-03

**Authors:** Jiaying Chen, Xuxuan Gao, Jingru Liang, Qiheng Wu, Linlin Shen, Yifeng Zheng, Yu Ma, Yuping Peng, Yan He, Jia Yin

**Affiliations:** 1 Department of Neurology, Nanfang Hospital, Southern Medical University, Guangzhou, Guangdong, China; 2 Comprehensive Medical Treatment Ward, Nanfang Hospital, Southern Medical University, Guangzhou, China; 3 Department of Neurosurgery, The Third Affiliated Hospital of Southern Medical University, Guangzhou, China; 4 Microbiome Medicine Center, Department of Laboratory Medicine, Zhujiang Hospital, Southern Medical University, Guangzhou, Guangdong, China; 5 Guangdong Provincial Clinical Research Center for Laboratory Medicine, Guangzhou, Guangdong, China; 6 State Key Laboratory of Organ Failure Research, Southern Medical University, Guangzhou, Guangdong, China; 7 Key Laboratory of Mental Health of the Ministry of Education, Guangzhou, Guangdong, China; The University of Hong Kong, Hong Kong, Hong Kong

**Keywords:** gut microbiota, dysbiosis, stroke, COVID-19, functional outcomes

## Abstract

Acute ischemic stroke (AIS) patients with active COVID-19 infection often have more severe symptoms and worse recovery. COVID-19 infection can cause gut microbiota dysbiosis, which is also a risk factor for poor outcomes in AIS patients. However, the association between gut microbiota and functional outcomes among AIS patients with COVID-19 infection has not been fully clarified yet. In this study, we performed 16S rRNA gene sequencing to characterize the gut microbial community among AIS patients with acute COVID-19 infection, AIS patients with post-acute COVID-19 infection, and AIS patients without COVID-19 infection. We found that AIS patients with acute COVID-19 experienced poorer recovery and significant gut dysbiosis, characterized by higher levels of *Enterobacteriaceae* and lower levels of *Ruminococcaceae* and *Lachnospiraceae*. Furthermore, a shorter time window (less than 28 days) between COVID-19 infection and stroke was identified as a risk factor for poor functional outcomes in AIS patients with COVID-19, and the enrichment of *Enterobacteriaceae* was indicated as a mediator in the relationship between infection time window and poor stroke outcomes. Our findings highlight the importance of early intervention after COVID-19 infection, especially by regulating the gut microbiota, which plays a role in the prognosis of AIS patients with COVID-19 infection.

**IMPORTANCE:**

The gut microbiota plays an important role in the association between respiratory system and cerebrovascular system through the gut-lung axis and gut-brain axis. However, the specific connection between gut bacteria and the functional outcomes of acute ischemic stroke (AIS) patients with COVID-19 is not fully understood yet. In our study, we observed a significant decrease in bacterial diversity and shifts in the abundance of key bacterial families in AIS patients with acute COVID-19 infection. Furthermore, we identified that the time window was a critical influence factor for stroke outcomes, and the enrichment of *Enterobacteriaceae* acted as a mediator in the relationship between the infection time window and poor stroke outcomes. Our research provides a new perspective on the complex interplay among AIS, COVID-19 infection, and gut microbiota dysbiosis. Moreover, recognizing *Enterobacteriaceae* as a potential mediator of poor stroke prognosis offers a novel avenue for future exploration and therapeutic interventions.

## INTRODUCTION

In the post-pandemic COVID-19 period, COVID-19 remains a threat to the healthcare systems with continuous emergence of multiple SARS-CoV-2 variants (1, 2). As of 22 December 2023, there are over 772 million confirmed cases and nearly 7 million deaths have been reported globally due to COVID-19 (3). Despite its primary manifestation as a pulmonary illness, COVID-19 exerts systemic effects on extra-respiratory organ systems, particularly the cardiocerebral vascular system (4–7). Infection with COVID-19 is associated with endothelial damage and hypercoagulability, increasing the risk of complications such as venous thrombosis (8), myocardial injury (9), and stroke (10, 11). The mean prevalence of acute stroke in COVID-19 patients is estimated at 1.5%, and these patients often experience more severe symptoms and worse recovery (12–14). However, the mechanisms of how pre-stroke COVID-19 infection influences stroke outcomes remain not fully understood.

The gastrointestinal tract is considered to be the largest immunological organ, and its resident microbiota can modulate host immune responses, defend against infections, and provide nutrients for metabolism (15–17). Infection with SARS-CoV-2 can downregulate the angiotensin-converting enzyme 2 (ACE2) receptor in the intestines, leading to gut microbiota dysbiosis (18–21). This dysbiosis, characterized by decreased microbial diversity, an enrichment of opportunistic pathogens, and a depletion of beneficial commensals (22–24), results in the translocation of bacterial toxins into the circulatory system and the exacerbation of inflammation (22, 25, 26). In addition, gut microbiota dysbiosis in COVID-19 patients can last for at least 30 days even after the clearance of SARS-CoV-2 infection (26).

Previous studies have demonstrated that gut microbiota dysbiosis is a risk factor for poor outcomes in acute ischemic stroke (AIS) patients (27, 28). The gut-brain axis, a bidirectional communication system, designates the gut microbiota as a primary mediator in this process (29). Despite this, limited data exist on the association between gut microbiota dysbiosis and COVID-19 infection among AIS patients. In this study, we investigated the gut microbiota of AIS patients with acute and post-acute COVID-19 infections, comparing them with AIS patients without COVID-19 infection. Additionally, we identified the infection time window as a risk factor for poor outcomes in AIS patients with COVID-19 infection and investigated the potential mediating role of gut microbiota in this relationship.

## RESULTS

### Characteristics of the study participants

**Fig 1:**
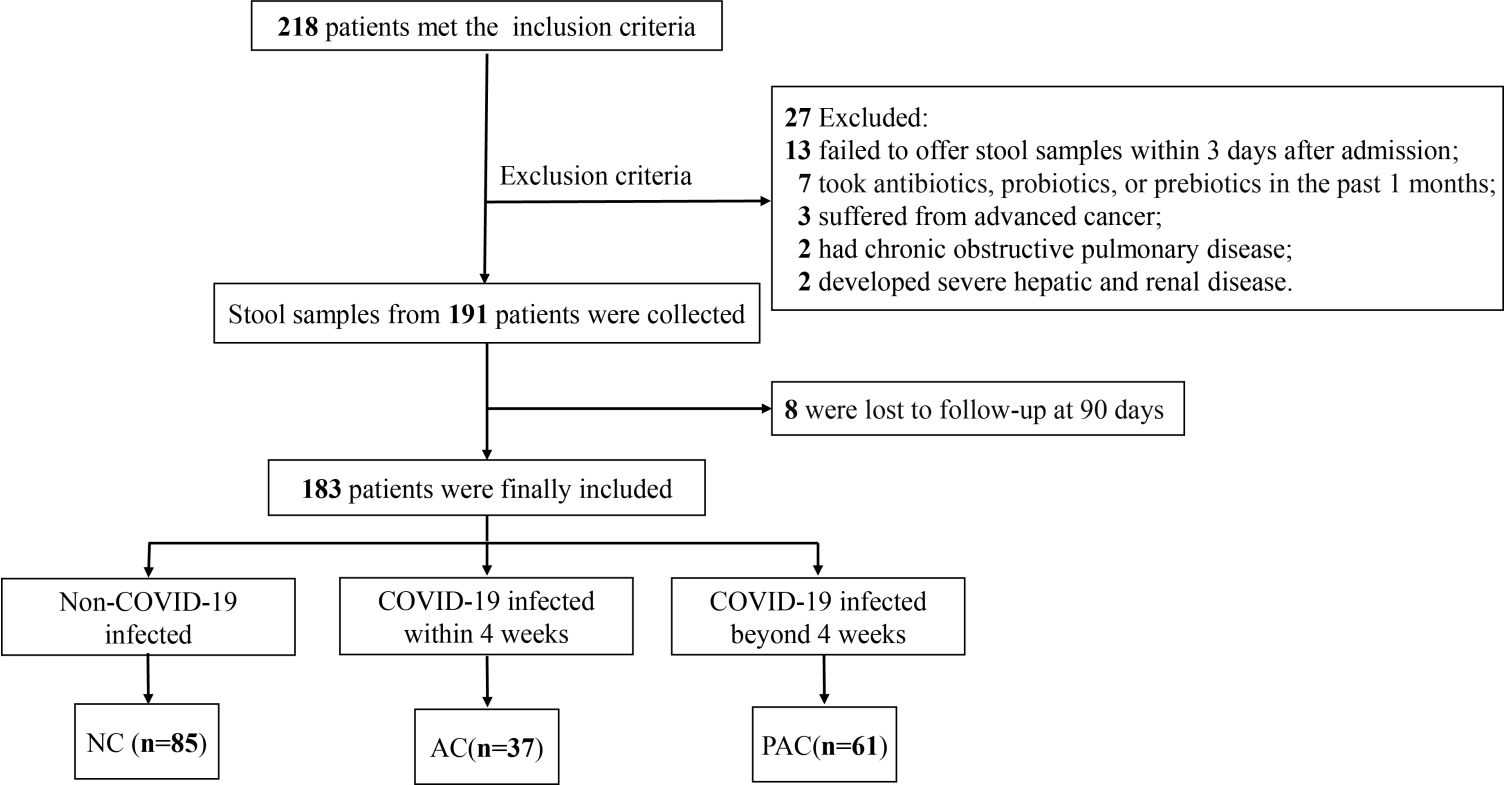
Flow chart of enrollment process. NC, non-COVID-19 group; AC, acute COVID-19 group; PAC, post-acute COVID-19.

**Fig 2:**
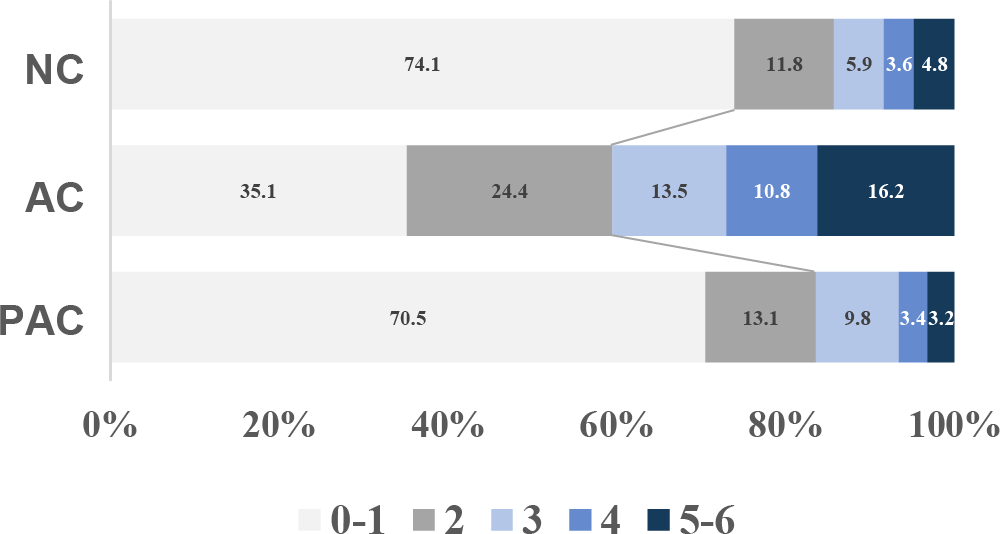
Distribution of modified Rankin scale score at 90-day follow-up in the three groups.

A total of 183 AIS patients were enrolled in the study, including 85 in the non-COVID-19 (NC) group, 37 in the acute COVID-19 (AC) group, and 61 in the post-acute COVID-19 (PAC) group (Fig. 1). There were no significant differences in the demographic factors [age, sex, body mass index (BMI)], risk factors (hypertension, diabetes, hyperlipidemia, atrial fibrillation, coronary heart disease, smoking, prior stroke), and Trial of Org10172 in Acute Stroke Treatment (TOAST) classification among the three groups (Table 1). Compared with the NC and PAC groups, the AC group had higher National Institutes of Health Stroke Scale (NIHSS) scores at admission and a greater prevalence of moderate to severe stroke. Moreover, the AC group showed significantly higher D-dimer levels and lower levels of hemoglobin (HGB) and albumin (ALB) when compared with the other two groups. The levels of interleukin-6 (IL-6) and C-reactive protein (CRP) were significantly increased in the AC group. At the 90-day follow-up, poor functional outcomes [modified Rankin scale (mRS) > 2] were identified in 40.5% of the AC group, in contrast to 14.1% in the NC group and 16.4% in the PAC group (*P* = 0.003; Table 1; Fig. 2). Our data showed that patients in the AC group had more severe symptoms along with a hypercoagulable and proinflammatory state and worse recovery.

**TABLE 1:** Characteristics of the study participants^*a*^

Characteristic	Total (*n* = 183)	NC (*n* = 85)	AC (*n* = 37)	PAC (*n* = 61)	*P* value
Demographics					
Age, years	63.0 (12.0)	64.0 (14.5)	63.0 (9.5)	60.0 (14.0)	0.481
Sex, male	130 (71.0)	58 (68.2)	26 (70.3)	46 (75.4)	0.639
BMI	23.8 (4.3)	23.8 (4.7)	23.7 (2.6)	23.9 (4.5)	0.844
Risk factors					
Hypertension	130 (71.0)	59 (69.4)	24 (64.9)	47 (77.0)	0.395
Diabetes mellitus	68 (37.2)	33 (38.8)	17 (45.9)	18 (29.5)	0.242
Hyperlipidemia	78 (42.6)	34 (40.0)	12 (32.4)	32 (52.2)	0.122
Atrial fibrillation	10 (5.5)	3 (3.5)	3 (8.1)	4 (6.6)	0.535
Coronary heart disease	13 (7.1)	5 (5.9)	3 (8.1)	5 (8.2)	0.836
Smoking	93 (50.8)	42 (49.4)	18 (48.6)	33 (54.1)	0.820
Prior stroke	48 (26.2)	22 (25.9)	13 (35.1)	13 (21.3)	0.321
NIHSS admission	2.0 (3.0)	2.0 (3.0)	3.0 (5.0)^*b*^^,^^*c*^	2.0 (4.0)	0.009^*d*^
Mild (NIHSS 0–4)	133 (72.7)	68 (80)	21 (56.8)^*b*^	44 (72.1)	0.030^*d*^
Moderate-severe (NIHSS ≥ 5)	50 (27.3)	17 (20)	16 (43.2)	17 (27.9)	
TOAST					0.062
Large artery atherosclerosis	124 (67.8)	63 (74.1)	26 (70.3)	35 (57.4)	
Cardioembolism	9 (4.9)	2 (3.5)	2 (5.4)	4 (6.6)	
Small vessel occlusion	13 (7.1)	6 (7.1)	2 (5.4)	5 (8.2)	
Other determined	15 (8.2)	7 (8.2)	4 (10.8)	4 (6.6)	
Undetermined	22 (12.0)	6 (7.1)	3 (8.1)	13 (21.3)	
Laboratory findings					
WBC, ×10^9^ /L	7.71 (3.39)	7.23 (3.46)	7.77 (3.21)	7.99 (3.74)	0.278
NEU, ×10^9^ /L	5.04 (2.61)	4.73 (2.60)	5.74 (2.59)	4.86 (3.02)	0.299
LYM, ×10^9^ /L	1.72 (1.03)	1.81 (0.87)	1.48 (0.71)^*b*^	1.76 (1.34)	0.048^*d*^
HGB, g/L	138.0 (21.00)	138.0 (18.00)	130.0 (22.00)^*b*^^,^^*c*^	141.0 (27.00)	0.017^*d*^
ALB, µmol/L	41.2 (7.50)	42.6 (6.80)	39.0 (8.35)^*b*^^,^^*c*^	42.2 (7.10)	0.011^*d*^
D-dimer, ng/mL	0.36 (0.55)	0.29 (0.39)	0.64 (0.63)^*b*^^,^^*c*^	0.36 (0.56)	0.005^*d*^
CRP, mg/L	2.56 (5.90)	2.04 (3.62)	3.92 (17.0)^*b*^	2.59 (9.39)	0.014^*d*^
IL-6, pg/mL	3.89 (7.64)	3.59 (3.21)	6.18 (14.03)^*b*^	5.84 (13.29)	0.019^*d*^
Outcomes					
Discharge mRS	1.0 (2.0)	1.0 (2.0)	1.0 (3.0)^*b*^	1.0 (1.0)	0.019^*d*^
Prognosis at 90 days					0.003^*d*^
Good (mRS 0–2)	146 (79.8)	73 (85.9)	22 (59.5)	51 (83.6)	
Poor (mRS 3–6)	37 (20.2)	12 (14.1)	15 (40.5)	10 (16.4)	

^*a*^ Continuous variables are expressed as medians (IQR), and categorical variables are expressed as frequencies (percentages). NC, non-COVID-19 group; AC, acute COVID-19 group; PAC, post-acute COVID-19. BMI, body mass index; WBC, white blood cell count; NEU, neutrophil count; LYM, lymphocyte count; HGB, hemoglobin; ALB, albumin; CRP, C-reactive protein; IL-6, interleukin-6.

^*b*^ *P* < 0.05 when acute COVID-19 group compared with non-COVID-19 group.

^*c*^ *P* < 0.05 when acute COVID-19 group compared with post-acute COVID-19 group.

^*d*^ *P* < 0.05, significant difference among the three groups.

### Differences in the composition of gut microbial communities among the AC, NC, and PAC groups

**Fig 3:**
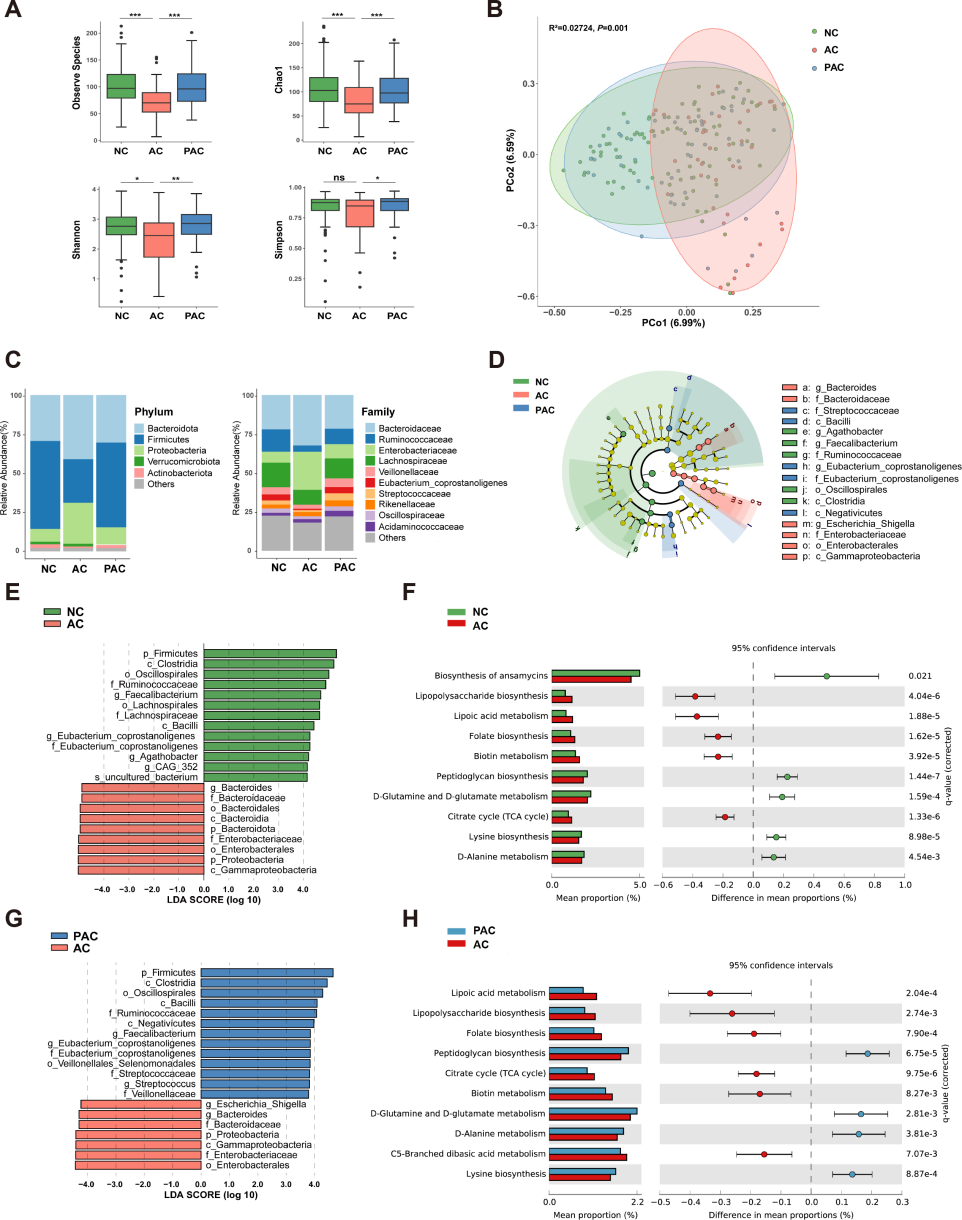
Comparisons of gut microbiota among the NC, AC, and PAC groups. (**A**) Alpha-diversity of gut microbiota among the three groups, including observed species, Chao1, Shannon, and Simpson index. (**B**) PCoA plot showing beta-diversity of gut microbiota composition. (**C**) Relative abundance of prevalent microbiota at phylum and family levels. (**D**) LEfSe analysis of the three groups. LEfSe analysis of (**E**) NC and AC groups, as well as (**G**) PAC and AC groups. Functional predictions of differential taxa between (**F**) NC and AC groups, as well as between (**H**) PAC and AC groups. ^*^P < 0.05, ^**^P < 0.01, and ^***^P < 0.001.

To investigate the association between gut dysbiosis and COVID-19 infection in AIS patients, we conducted 16S rRNA sequencing on fecal samples from the three groups. The AC group showed significantly lower alpha-diversity, including observed species, Chao1, Shannon index, and Simpson, compared to the NC and PAC groups (Fig. 3A). Additionally, the PCoA plot demonstrated significant differences in beta-diversity between the AC group and the other two groups (R2: 0.02724; *P* = 0.001; Fig. 3B). At the phylum level, the AC group showed an increased abundance of *Proteobacteria* and a decreased abundance of *Firmicutes* compared to the other groups. At the family level, the AC group showed a higher abundance of *Enterobacteriaceae* and a reduced abundance of *Ruminococcaceae* and *Lachnospiraceae* (Fig. 3C; Fig. S1A). Moreover, linear discriminant analysis effect size (LEfSe) analysis highlighted significant enrichment of *Bacteroidaceae* and *Enterobacteriaceae*, along with lower relative abundances of *Ruminococcaceae* and *Eubacterium coporostanoligenes* at the family level in the AC group compared to the NC and PAC groups (Fig. 3D and E). No significant difference was observed between the NC and PAC groups, except for the relative abundances of *Negativicutes* at the class level (Fig. S1B). Further PICRUSt analysis identified an enrichment of metabolites and pathways related to lipopolysaccharide biosynthesis, lipoic acid metabolism, folate biosynthesis, biotin metabolism, folate biosynthesis, and the citrate cycle in the AC group compared to the NC and PAC groups (Fig. 3F). Therefore, these data showed significant gut dysbiosis in the AC group.

### Associations between gut dysbiosis and 90-day functional outcomes in AIS patients with COVID-19 infection

Of the 98 AIS patients with COVID-19 infection (i.e., the AC and PAC groups), 25.5% (25/98) had poor functional outcomes at the 90-day follow-up. These patients had significantly higher NIHSS scores at admission, elevated D-dimer levels, and lower BMI. Furthermore, we observed that patients with poor functional outcomes had a shorter time window between COVID-19 infection and stroke, fewer vaccinations, and more gastrointestinal symptoms (Table 2). Logistic regression analysis showed that a shorter time window (less than 28 days) between COVID-19 infection and stroke was a significant risk factor [odds ratio (OR) = 3.63; 95% confidence interval (CI), 1.09–12.08; *P* = 0.036] for poor stroke outcomes, even after adjusting for BMI, NIHSS scores at admission, doses of vaccines, and gastrointestinal symptoms (Table 3 Model 1).

**TABLE 2:** Characteristics of patients with different functional outcomes among AIS patients with COVID-19 infection^*a*^

Characteristic	Total, *n* = 98	Good, *n* = 73	Poor, *n* = 25	*P* value
Age, years	63 (12)	61 (14)	64 (9)	0.083
Sex, male	72 (73.5)	55 (75.3)	17 (68.0)	0.475
BMI	23.79 (3.60)	24.2 (3.75)	21.5 (4.60)	<0.001^*b*^
Comorbidities				
Hypertension	71 (72.4)	53 (72.6)	18 (72.0)	0.954
Diabetes mellitus	35 (35.7)	24 (32.9)	11 (44.0)	0.319
Hypercholesterolemia	44 (44.9)	36 (49.3)	8 (32.0)	0.135
Atrial fibrillation	7 (7.1)	3 (4.1)	4 (16.0)	0.047^*b*^
Coronary heart disease	8 (8.2)	4 (5.5)	4 (16.0)	0.099
Smoking	51 (52.0)	37 (50.7)	14 (56.0)	0.648
Prior stroke	26 (26.5)	19 (26.0)	7 (28.0)	0.848
NIHSS admission	3.0 (4.0)	2.0 (2.0)	7.0 (8.0)	<0.001^*b*^
Mild (NIHSS 0–4)	65 (66.3)	58 (79.5)	7 (28)	<0.001^*b*^
Moderate-severe (NIHSS ≥5)	33 (33.7)	15 (20.5)	18 (72)	
TOAST				
Large artery atherosclerosis	61 (62.2)	46 (63.0)	15 (60.0)	0.993
Cardioembolism	6 (6.1)	3 (4.1)	3 (12.0)	
Small vessel occlusion	7 (7.1)	6 (8.2)	1 (4.0)	
Other determined	8 (8.2)	5 (6.8)	3 (12.0)	
Undetermined	16 (16.3)	13 (17.8)	3 (12.0)	
Laboratory findings				
WBC, ×10^9^ /L	7.91 (3.44)	7.96 (3.40)	7.86 (3.85)	0.690
NEU, ×10^9^ /L	5.29 (2.84)	5.24 (2.80)	5.40 (3.07)	0.473
LYM, ×10^9^ /L	1.61 (1.17)	1.68 (1.25)	1.49 (0.73)	0.408
HGB, g/L	136.0 (22.5)	136.0 (22.5)	134.0 (37.5)	0.220
ALB, µmol/L	40.65 (8.50	41.10 (7.40)	39.70 (10.3)	0.173
TG, mmol/L	1.45 (1.06)	1.45 (1.41)	1.44 (0.74)	0.303
D-dimer, ng/mL	0.45 (0.55)	0.39 (0.50)	0.73 (0.80)	0.023^*b*^
CRP, mg/L	3.13 (10.38)	2.28 (7.47)	7.09 (39.23)	0.059
IL-6, pg/L	6.01 (12.65)	5.54 (10.60)	7.14 (21.66)	0.530
COVID-19 infection				
Infection time window, days	43.5 (54)	51.0 (53.0)	28.0 (50.0)	0.013^*b*^
Doses of vaccines	3.0 (1.0)	3.0 (1.0)	2.0 (3.0)	0.028^*b*^
Respiratory symptoms	87 (88.8)	66 (90.4)	21 (84.0)	0.383
Gastrointestinal symptoms	35 (35.7)	21 (28.8)	14 (56.0)	0.014^*b*^

^*a*^ Continuous variables are expressed as medians (IQR), and categorical variables are expressed as frequencies (percentages). Respiratory symptoms: fever, cough, phlegm, sore throat, nasal congestion, or dyspnea. Gastrointestinal symptoms: abdominal pain, diarrhea, nausea, vomiting, or loss of appetite.

^*b*^ *P* < 0.05, significant difference.

**TABLE 3:** Univariate and multivariate regression analysis of factors associated with poor functional outcomes in AIS patients with COVID-19 infection^*a*^

Variable	Univariate analysis		Multivariate analysis		Multivariate analysis	
	OR (95% CI)	*P* value	Model 1 OR (95% CI)	*P* value	Model 2 OR (95% CI)	*P* value
Clinical indices						
Age	1.05 (0.99–1.10)	0.054	–^*f*^	–	–	–
BMI	0.71 (0.58–0.87)	0.001	0.70 (0.55–0.89)	0.005^*e*^	0.74 (0.59–0.93)	0.009^*e*^
Atrial fibrillation	4.44 (0.92–21.46)	0.063	–	–	–	–
NIHSS admission^*b*^	9.94 (3.51–28.17)	<0.001	5.35 (1.64–17.52)	0.008^*e*^	6.54 (1.93–22.21)	0.003^*e*^
Infection time window^*c*^	3.48 (1.35–8.93)	0.010	3.63 (1.09–12.08)	0.036^*e*^	–	–
Doses of vaccines	0.60 (0.40–0.90)	0.014	0.63 (0.37–1.06)	0.082	–	–
Gastrointestinal symptoms	2.51 (0.99–6.37)	0.016	1.70 (0.51–5.66)	0.387	–	–
D-dimer	1.37 (0.99–1.87)	0.052	–	–	–	–
Family^*d*^						
*Bacteroidaceae*	0.97 (0.94–0.99)	0.018^*e*^	–	–	0.98 (0.95–1.01)	0.257
*Enterococcaceae*	1.02 (0.98–1.07)	0.368	–	–	–	–
*Enterobacteriaceae*	1.04 (1.02–1.07)	<0.001^*e*^	–	–	1.04 (1.01–1.07)	0.006^*e*^

^*a*^ Model 1: adjusted clinical indices with *P* < 0.05 in univariate analysis. Model 2: adjusted for BMI and NIHSS scores at admission.

^*b*^ NIHSS admission: NIHSS score 0–4 vs NIHSS score ≥5.

^*c*^ Infection time window: infection days >28 vs infection days ≤ 28.

^*d*^ Relative abundance (%) of gut microbiota at the family levels.

^*e*^ *P* < 0.05, significant difference.

^*f*^ –, not included in the multivariate analysis model.

**Fig 4:**
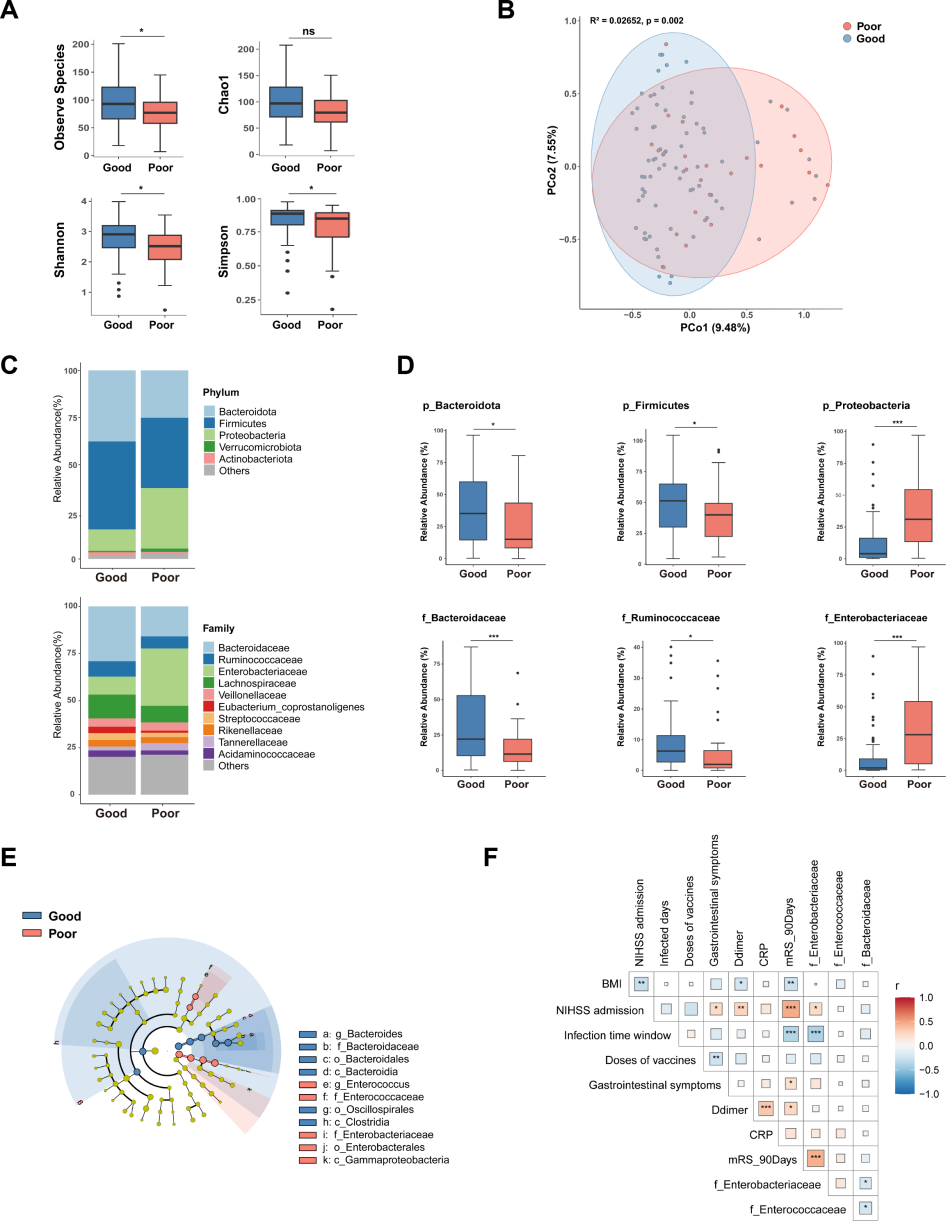
Gut microbiota of patients with different functional outcomes among AIS patients with COVID-19 infection. (**A**) Alpha-diversity of gut microbiota. (**B**) PCoA plot showing beta-diversity of gut microbiota composition. (**C**) Relative abundances of prevalent microbiota at phylum and family levels. (**D**) Relative abundance of *Bacteroidota*, *Firmicutes*, and *Proteobacteria*a at phylum levels and *Bacteroidaceae*, *Enterobacteriaceae*, and *Ruminococcaceae* at family levels. (**F**) LEfSe analysis of the two groups. (**G**) Spearman correlation analysis of the association of intestinal microbiota with clinical characteristics. ^*^P < 0.05, ^**^P < 0.01, and ^***^P < 0.001.

To further explore the correlation between gut dysbiosis and poor functional outcomes in AIS patients with COVID-19 infection, we compared the gut microbiota composition in patients with different outcomes. We found that patients with poor functional outcomes showed a significant decrease in alpha-diversity (Fig. 4A), along with significant variations in beta-diversity when compared with the patients with good functional outcomes (Fig. 4B). In terms of taxonomic distribution, patients with poor functional outcomes showed higher levels of *Enterobacteriaceae* and lower levels of *Ruminococcaceae* and *Bacteroidaceae* at the family level (Fig. 4C and D). Further LEfSe analysis showed significant enrichment of *Enterococcaceae* and *Enterobacteriaceae* at the family level in patients with poor functional outcomes (Fig. 4E). Moreover, logistic regression analysis showed that the enrichment of *Enterobacteriaceae* was a risk factor (OR = 1.04; 95% CI, 1.01–1.07; *P* = 0.006) for poor stroke outcomes, after adjusting for BMI and NIHSS scores at admission (Table 3 Model 2).

### *Enterobacteriaceae* mediated the relationship between the time window from COVID-19 infection to stroke and poor functional outcomes

**Fig 5:**
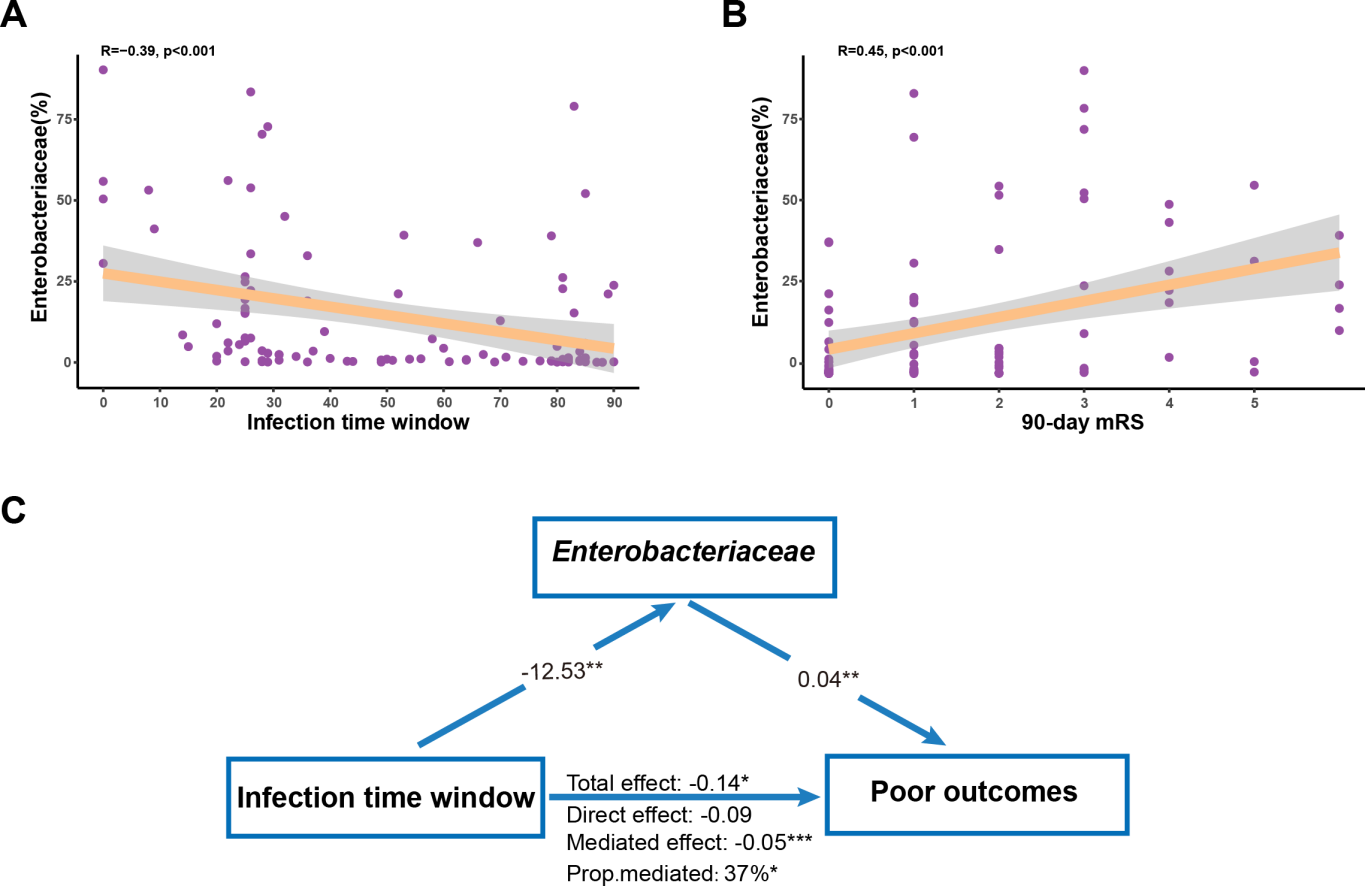
*Enterobacteriaceae* mediated the relationship between infection time window and poor functional outcomes. Scatter plots and Spearman correlation coefficients between (**A**) the enrichment of *Enterobacteriaceae* and infection time window, as well as (**B**) the enrichment of *Enterobacteriaceae* and 90-day mRS. (**C**) Mediation analyses testing *Enterobacteriaceae* as mediators between infection time window and poor functional outcomes. ^*^P < 0.05, ^**^P < 0.01, and ^***^P < 0.001.

We performed a Spearman correlation analysis to investigate the association between gut microbiota and clinical characteristics (Fig. 4F). We found a significant negative correlation (R = −0.39; *P* < 0.001) between the enrichment of *Enterobacteriaceae* and the infection time window (Fig. 4F and 5A). Additionally, a positive correlation (R = −0.45; *P* < 0.001) was observed between the enrichment of *Enterobacteriaceae* and 90-day mRS scores (Fig. 4F and 5B). To further explore whether the enrichment of *Enterobacteriaceae* mediated the relationship between the infection time window and poor stroke outcomes, we conducted mediation analysis. Our results indicated that the enrichment of *Enterobacteriaceae* acted as a mediator (mediated effect = −0.05; *P <* 0.001) in the relationship between the infection time window and poor stroke outcomes, even with adjustments for BMI, NIHSS scores at admission, doses of vaccines, and gastrointestinal symptoms (Fig. 5C; Table S1).

## DISCUSSION

Pre-stroke respiratory infections have been associated with increased morbidity and mortality in stroke (30, 31). Previous studies have demonstrated that gut dysbiosis played an important role in both the central nervous system and the respiratory system (32, 33). However, the relationship between COVID-19 infection, stroke, and gut microbiota remains unclear. In this study, we employed 16S rRNA sequencing to evaluate the association between gut dysbiosis and COVID-19 infection in AIS patients. Our results showed that AIS patients with acute COVID-19 infection had a worse recovery and significant gut dysbiosis. A shorter time window after infection emerged as a risk factor for poor functional outcomes in AIS patients with COVID-19 infection, where the enrichment of *Enterobacteriaceae* acted as a mediator.

Acute COVID-19 infection can cause endothelial damage and a prothrombotic condition, raising the risk of stroke and other vascular disorders (6, 9). Our study observed that AIS patients with acute COVID-19 had more severe stroke symptoms and experienced poorer recovery, consistent with previous studies (34, 35). Additionally, elevated levels of D-dimer, IL-6, and CRP in AIS patients with acute COVID-19 suggest that pre-stroke COVID-19 infection may contribute to increased secondary inflammation post-stroke onset. Acute infection as a trigger of stroke has a greater potential influence within a shorter time window (36, 37). Notably, we observed no significant difference in stroke symptoms and prognosis between PAC and NC groups, indicating a time-limited impact of infection on stroke recovery. Moreover, our logistic regression analysis identified a shorter infection time window as a risk factor for poor functional outcomes in AIS patients with COVID-19. Therefore, infection time window is a critical factor influencing the subsequent stroke prognosis.

Although SARS-CoV-2 primarily affects the respiratory system, gastrointestinal symptoms are also commonly observed in COVID-19 patients. In this study, 35.7% (35/98) of patients experienced at least one gastrointestinal symptom during COVID-19 infection, consistent with a prevalence of 15–69% reported in the literature (38–40). Previous studies have shown that COVID-19 patients with gastrointestinal symptoms had a significantly higher rate of severe illness (40, 41). Notably, we found that patients with poor stroke outcomes were more likely to experience gastrointestinal symptoms during the infection. These gastrointestinal symptoms may suggest an increase in intestinal permeability or dysbiosis of gut microbiota (42).

In the present study, we observed a significantly higher abundance of opportunistic pathogenic bacteria (e.g., *Proteobacteria*, *Enterobacteriaceae*) in AIS patients with acute COVID-19. Additionally, the gut microbiota of these patients exhibited an enrichment in lipopolysaccharide biosynthesis pathway, a key mechanism that contributes to the aggravation of systemic inflammation and cerebral infarction due to post-stroke gut dysbiosis as we reported previously (28). Intriguingly, the gut microbiota composition and clinical prognosis of AIS patients with post-acute COVID-19 infection were similar to those without COVID-19 infection. This suggested that the gut microbiota may gradually recover or stabilize as the infection window extends, and its impact on stroke prognosis may correspondingly diminish.

COVID-19 infection can lead to alterations in the gut microbiota composition, contributing to inflammation and disease aggravation (23, 24, 26, 43, 44). Potential mechanisms by which COVID-19 infection triggers gut microbiota dysbiosis include the activation of pattern recognition receptors, downregulation of ACE2 expression facilitating pathogenic bacterial growth, and direct bacterial infection (45). The overgrowth of opportunistic pathogens could breach the intestinal barrier, entering the circulatory system, and exacerbating the systemic inflammatory response in COVID-19 patients (43, 44, 46). Our study showed that microbial community dysbiosis, particularly the enrichment of *Enterobacteriaceae*, was associated with a poor stroke prognosis in AIS patients with COVID-19 infection. The overgrowth of *Enterobacteriaceae* could accelerate systemic inflammation and exacerbate brain infarction (28). Moreover, mediation analysis found that *Enterobacteriaceae* functioned as a mediator in the relationship between the infection time window and poor stroke outcomes. These data indicated that the gut microbiota could be therapeutic targets to enhance the recovery of AIS patients with acute COVID-19 infection.

Despite providing valuable insights into the association between gut dysbiosis and functional outcomes in AIS patients with COVID-19 infection, our study has several limitations. Firstly, the relatively small sample size in this observational study may introduce potential bias. Secondly, functional enrichment analysis was conducted based on 16S rRNA sequencing data. To better illustrate correlations between specific microbiota and metabolites, a further integrated multi-omics study could be performed. Thirdly, our observational study could not determine the causal relationships between gut dysbiosis and functional outcomes. Further studies are needed to investigate the role and mechanism of microbiota homeostasis in AIS patients with COVID-19 infection.

In conclusion, our study revealed that the time window from COVID-19 infection to stroke was a risk factor for stroke prognosis, and the gut microbiota played a crucial mediating role in COVID-19 infection and stroke outcomes. Early intervention of gut microbiota after COVID-19 infection may help to improve the prognosis of AIS patients with COVID-19 infection.

## MATERIALS AND METHODS

### Study design and participants

This prospective observational cohort study was conducted at the Department of Neurology, Nanfang Hospital, Southern Medical University, and received approval from the Ethics Committee of Nanfang Hospital, Southern Medical University (NFEC-2020-169). Written informed consent was obtained from all study participants. Patients hospitalized with AIS from September 2022 to March 2023 were recruited. Inclusion criteria were as follows: (i) age greater than 18 years; (ii) admission within 7 days of ischemic stroke onset; and (iii) a reverse transcriptase-polymerase chain reaction (PCR) assay for SARS-CoV-2 in nasopharyngeal swabs before admission. Exclusion criteria were as follows: (i) failed to offer stool samples within 3 days after admission; (ii) use of antibiotics, prebiotics, or probiotics used within 1 month before admission; (iii) history of chronic obstructive pulmonary disease; (iv) advanced cancer; (v) severe hepatic and renal disease; and (vi) lost to follow-up at 90 days. Patients with no symptoms of SARS-CoV-2 infection and tested negative for SARS-CoV-2 RNA were considered as the non-COVID-19 group. Acute COVID-19 typically persists for less than 4 weeks after the onset of symptoms, after which replication-competent SARS-CoV-2 has not been identified (47, 48). Patients with COVID-19 infection were categorized into the acute COVID-19 group (within 4 weeks from the onset of symptoms) and post-acute COVID-19 group (beyond 4 weeks from the onset of symptoms).

### Clinical data and sample collection

The clinical data, including age, sex, body mass index, vascular risk factors (hypertension, diabetes mellitus, hyperlipidemia, atrial fibrillation, coronary heart disease, smoking, and prior stroke), NIHSS score, TOAST classification, and relevant laboratory results were collected and determined by experienced neurologists at admission. Information related to COVID-19 infection, such as the time window between COVID-19 infection and stroke, vaccine dosages, symptoms involving the respiratory and gastrointestinal systems, were also documented. Modified Rankin scale score at discharge and at 90-day follow-up was obtained prospectively. Fecal samples were collected within 3 days after admission and stored at −80°C until DNA extraction was performed.

### 16S rRNA sequencing

Bacterial genomic DNA was extracted from fecal samples using the QIAamp Power-Fecal Pro DNA Kit (QIAGEN, USA) following the manufacturer's instructions. The V4 hypervariable region of the 16S rRNA gene was amplified by PCR using barcoded primers: V4F (5′GTGTGYCAGCMGCCGGTAA3′) and V4R (5′CCGGACTACNVGGGTWTCTAAT3′). Subsequently, all PCR amplicons were combined and sequenced on the Illumina NovaSeq 6000 platform.

Microbiota data analysis was conducted using QIIME 2, including demultiplexing, primer removal, quality control, and taxonomy assignment (49). Briefly, the length- and quality-filtered reads were binned into amplicon sequence variants using DADA2 (50). Taxonomic assignment was performed using the q2-feature-classifier plugin (51) based on the Silva 138 16S rRNA reference database (52). Alpha-diversity, including observed species, Shannon index, Simpson index, and Chao1 index, was calculated. Beta diversity was estimated by computing Bray-Curtis distances, followed by their utilization in principal coordinate analysis (PCoA). Differential taxa between groups were determined using LEfSe with an linear discriminant analysis (LDA) score threshold of 4. Additionally, functional prediction of 16S rRNA sequencing data was performed using PICRUSt2 software (53) against the Kyoto Encyclopedia of Genes and Genomes (KEGG) database (54).

### Statistical analysis

Statistical analyses were performed using IBM SPSS 26.0 software and R version 4.2.1. Categorical variables and continuous variables are presented as frequencies (percentages) and medians (interquartile range, IQR), respectively. Clinical data among groups were compared using the Kruskal-Wallis test, the Mann-Whitney U-test, and the Pearson χ^2^ test. The OR with a 95% CI was calculated using multivariate logistic regression analyses to explore clinical characteristics associated with the 90-day poor functional outcomes (mRS > 2). The correlation between specific microbiota and clinical characteristics was analyzed by Spearman's rank correlation. Restricted cubic spline was used to illustrate the association between the time window from COVID-19 infection to stroke and poor functional outcomes. Mediation analysis was performed to determine whether the association between the time window from COVID-19 infection to stroke and poor stroke outcomes was mediated by the enrichment of *Enterobacteriaceae*. A value of *P* < 0.05 was considered statistically significant.

## Data Availability

The raw sequence data reported in this paper have been deposited in the Genome Sequence Archive (55) in National Genomics Data Center (56), China National Center for Bioinformation/Beijing Institute of Genomics, Chinese Academy of Sciences (GSA-Human: HRA006620) that are publicly accessible at https://bigd.big.ac.cn/gsa-human/browse/HRA006620. Additional data are available upon reasonable request and contacting the correspondent authors. The Strengthening the Organizing and Reporting of Microbiome Studies (STORMS) checklist is available at https://doi.org/10.5281/zenodo.10822221.

## SUPPLEMENTAL MATERIAL

Supplemental Data msystems.00185-24-s0001.xlsx Additional data.

Supplemental Information msystems.00185-24-s0002.docx Fig. S1 and Table S1.

ASM does not own the copyrights to Supplemental Material that may be linked to, or accessed through, an article. The authors have granted ASM a non-exclusive, world-wide license to publish the Supplemental Material files. Please contact the corresponding author directly for reuse.
