# Supplementary material for: Association between gut microbiota dysbiosis and poor functional outcomes in acute ischemic stroke patients with COVID-19 infection
Source: mSystems. 2024 May 3;9(6):e00185-24. doi: 10.1128/msystems.00185-24 (PMC11237522; doi:10.1128/msystems.00185-24)
Supplement: Supplemental Information — Fig. S1 and Table S1. [file msystems.00185-24-s0002.docx]

**Supplementary Information**

**Association between gut microbiota dysbiosis and poor functional outcomes in acute ischemic stroke patients with COVID-19 infection**

Jiaying Chen, Xuxuan Gao, Jingru Liang, Qiheng Wu, Linlin Shen, Yifeng Zheng, Yu Ma, Yuping Peng, Yan He, Jia Yin

**Supplementary Figure**


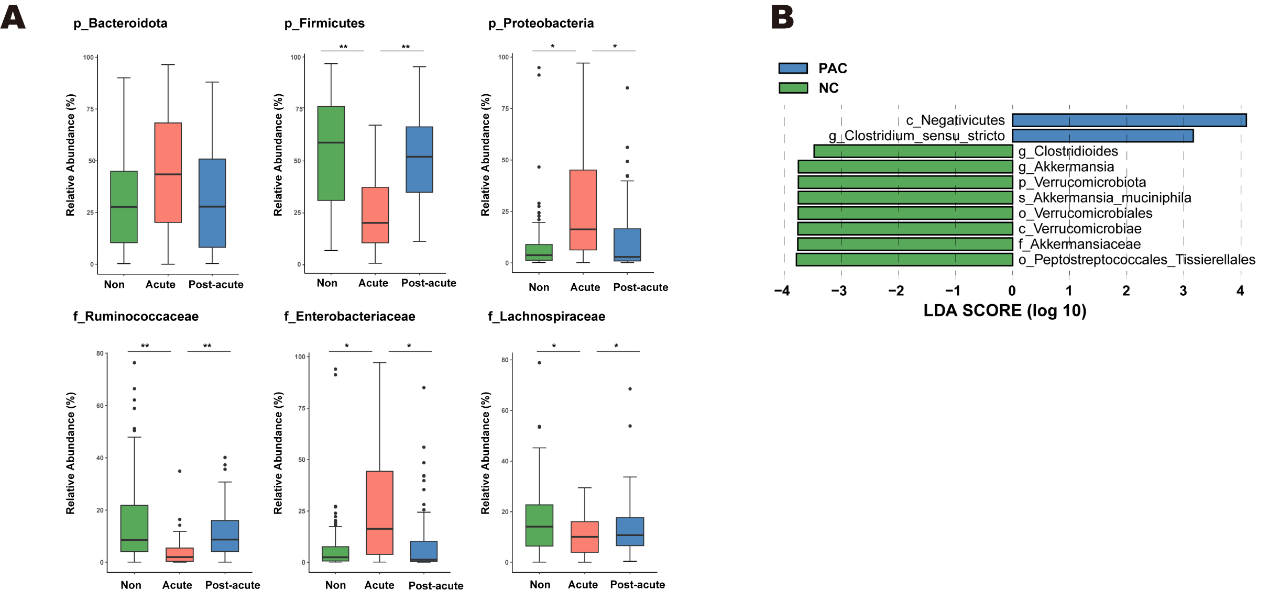


**Figure S1.** Comparisons of gut microbiota among the NC group, AC group, and PAC group. (A) Relative abundance of *Bacteroidota*, *Firmicutes*, and *Proteobacteria*a at phylum levels, and *Ruminococcaceae*, *Enterobacteriaceae* and *Lachnospiraceae* at family levels. (**C**) LEfSe analysis of PAC group and NC group. ^*^*P* < 0.05, ^**^*P* < 0.01, and ^***^*P* < 0.001.

**Supplementary Table**

**Table S1.** Mediation analyses testing *Enterobacteriaceae* as mediators between COVID-19 infected days and poor functional outcomes

| **Path** | **Effect Estimate** | **95%CI** | ***P* value** |
| --- | --- | --- | --- |
| a (X→M) | -12.53 | (-21.55, -3.51) | 0.007^*^ |
| b (M→Y) | 0.04 | (0.02, 0.08) | 0.005^*^ |
| c (total effect: X→Y) | -0.14 | (-0.44, 0.02) | 0.036^*^ |
| c’ (direct effect: X→Y) | -0.09 | (-0.02, 0.02) | 0.192 |
| ab(mediated effect: X→M→Y) | -0.05 | (-0.20, -0.02) | < 0.001^*^ |
| Proportion mediated effect | 0.37 | (0.40, 1.96) | 0.036^*^ |

Model adjusted for BMI, NIHSS at admission, doses of vaccines, and gastrointestinal symptoms.X: Infection time window (infection days > 28 VS infection days ≤ 28), M: *Enterobacteriaceae* (Relative abundance, %), Y: poor functional outcomes (90-day mRS 3-6).

^*^ *P* < 0.05, significant difference.
